# Blocking REDD1/TXNIP Complex Ameliorates HG-Induced Renal Tubular Epithelial Cell Apoptosis and EMT through Repressing Oxidative Stress

**DOI:** 10.1155/2022/6073911

**Published:** 2022-09-21

**Authors:** Lin Mu, Nan Chen, Yakun Chen, Zhifen Yang, Huandi Zhou, Shan Song, Yonghong Shi

**Affiliations:** ^1^Department of Pathology, Hebei Medical University, Shijiazhuang 050000, China; ^2^Hebei Key Laboratory of Kidney Disease, Shijiazhuang 050000, China; ^3^Department of Nephrology, Second Hospital of Hebei Medical University, Shijiazhuang 050000, China

## Abstract

Diabetic nephropathy (DN) has become the most common secondary kidney disease causing end-stage renal disease (ESRD). Nevertheless, the underlying mechanisms responsible for DN remain largely unknown. Regulated in development and DNA damage response 1 (REDD1) is a prooxidative molecule known to contribute to diabetes mellitus and its complications. However, it has not been previously examined whether and how REDD1 can further drive renal tubular epithelial cell (RTEC) apoptosis and epithelial-to-mesenchymal transition in DN. The expression of REDD1 was elevated in the kidneys of DN patients and diabetic mice in this study. By generating the DN model in REDD1 knockout mice, we demonstrated that REDD1 deficiency significantly improved apoptosis and EMT in diabetic mice. *In vitro* experiments showed that REDD1 generation was induced by high glucose (HG) in HK-2 cells. Similarly, the transfection of REDD1 siRNA plasmid also suppressed HG-induced apoptosis and EMT. Furthermore, we discovered that the inhibition of REDD1 suppressed the expression of Nox4-induced HG and reactive oxygen species (ROS) synthesis in HK-2 cells. In addition, HG could induce endogenous REDD1 and TXNIP to form a powerful complex. In summary, our findings demonstrate that blocking the REDD1/TXNIP complex can prevent HG-induced apoptosis and EMT by inhibiting ROS production, highlighting REDD1 as a valuable therapeutic priority site for DN.

## 1. Introduction

Diabetic nephropathy (DN) is secondary to diabetes mellitus [1]. As known to all, the prevalence of DN is approximately 30%–40% in diabetic patients [2]. DN accounts for approximately 40% of end-stage renal disease (ESRD) [3]. It is responsible for severe morbidity and mortality and eventually the need for renal replacement therapy. However, DN is still a clinically meaningful issue, and a better understanding of its underlying mechanisms holds the promise of identifying new therapeutic targets.

It has been well-grounded that renal tubular epithelial cell (RTEC) injury is a critical mechanism in the pathophysiology of DN [4]. Previous experiments of our research group have confirmed that high glucose- (HG-) induced RTEC apoptosis and epithelial-to-mesenchymal transition (EMT) were the main target and epicenter of the pathogenesis of DN [5, 6]. Moreover, reactive oxygen species (ROS) plays an important role in mediating DN [7]. In RTEC, HG may enhance intracellular ROS and promote RTEC apoptosis and EMT [8, 9]. Thus, reducing the generation of ROS may be protective against the kidney injury connected with DN.

Regulated in development and DNA damage response 1 (REDD1) is a newly discovered target gene for hypoxia-inducible factor-1, which is widely underexpressed in normal human tissues [10]. In some investigations, REDD1 is shown to be a quickly upregulated gene under a range of cellular stressors, including hypoxia, heat shock, and DNA damage [11]. Abnormal expression of REDD1 involves the occurrence of a multitude of illnesses, such as osteoarthritis, smoking-induced lung injury, Alzheimer's disease, and tumors [12, 13]. In addition, REDD1 is robustly increased by diabetes [14]. Research evidence suggests that the overexpression of REDD1 is important in the development of diabetic myopathy and diabetic retinopathy [15, 16]. Both nephropathy and retinopathy are microvascular complications of diabetes. It has been reported that 1,25(OH)_2_D_3_ can inhibit mesangial cell proliferation through REDD1 to improve the occurrence of early DN [17]. However, virtually, nothing is well known about the effect of REDD1 in the pathogenesis of DN.

Operating as a mammalian target of rapamycin (mTOR) endogenous inhibitor, REDD1 is an essential regulator of cell proliferation, oxidative stress, apoptosis, mitochondrial, and endoplasmic reticulum function [18, 19]. REDD1 has also been associated with ROS production [20]. Studies have found that the inhibition of REDD1 reduced lipopolysaccharide-induced oxidative stress and apoptosis in the vascular endothelial cell, which is independent of mTOR [21]. However, there is little knowledge on whether and how REDD1 may regulate oxidative stress and contribute to its role in RTEC apoptosis and EMT. Hence, the goal of this study is to see how REDD1 regulates HG-induced apoptosis and EMT and its underlying mechanism(s).

## 2. Materials and Methods

### 2.1. Human Renal Specimens

From 2018 to 2020, 40 patients were enrolled from the Department of Nephrology at Hebei Medical University's Second Hospital. Twenty samples of normoglycemic and type 2 diabetic kidney tissues without a history of DN were obtained from nephrectomies conducted to treat renal cancer as normal control. We obtained written informed consent from all patients, and patients' clinical and laboratory parameters were collected from medical records. Histopathological assessment was performed in a blinded manner by two pathologists and classified according to the pathologic presentation classification of DN. Hematoxylin and eosin (HE), periodic acid–Schiff (PAS), and immunohistochemical staining were performed on kidney tissues fixed with 4% formaldehyde.

### 2.2. Animal Models

The experimental protocols were performed in accordance with the guidelines from the Ethics Review Committee for Animal Experimentation of Hebei Medical University. All mice were kept in a pathogen-free environment. The dark/light cycle was adjusted to 12:12 h in their growth habitat.

#### 2.2.1. STZ Mice

Ten-week male C57BL/6J mice (30–40 g) were obtained from Beijing HFK Bioscience Co., Ltd. (Beijing, China). After 7 days of adaptation, 10 C57BL/6J mice were randomly divided into two groups: a control group (five mice) and a streptozotocin (STZ) group (five mice). Mice in the STZ group were given a 50 mg/kg STZ injection intraperitoneally for 5 days [22, 23]. Mice's blood glucose levels of more than 16.7 mmol/L for 3 days in a row were diagnosed as diabetic.

#### 2.2.2. C57BL/ks db/db Mice

Fifteen 8-week male C57BL/ks db/db mice (40–50 g) with genetic type 2 diabetes and five healthy db/m male mice (26–28 g) were purchased from the Model Animal Research Center of Nanjing University (Nanjing, China). Fifteen db/db mice were divided into three groups at random: db/db group (*n* = 5), db/db + blank group (*n* = 5), and db/db + REDD1shRNA group (*n* = 5). Five control mice served as the db/m group. In order to confirm the protective role of REDD1 inhibition on the kidney in vivo, 5 db/db mice were renally injected with 50 *μ*l 1 × 10^∧^12 infective units of HBAAV2/9-m-REDD1 shRNA-LUC into the kidneys at 8 weeks. In the meantime, the db/db + blank group was injected with the same dose of HBAAV2/9-LUC NC, and db/db group were injected with isometric saline [24, 25].

### 2.3. Cell Culture and Transfection

HK-2 cells from American Type Culture Collection were incubated in DMEM-F12 containing 10% fetal bovine serum, 100 U/ml penicillin, and 100 *μ*g/ml streptomycin at 37°C in a 5% CO_2_ atmosphere. HK-2 cells were respectively cultured in 5.6 mM normal glucose (NG), 30 mM HG, NG plus 24.4 mM mannitol as osmotic control, HG plus 5 mM NAC, and HG plus 100 nM Tempol [23, 26]. Transfection of cells with REDD1 siRNA and TXNIP siRNA by EndoFectin Max cells or their negative control by EndoFectin Max in accordance with the manufacturer's package recommendations. Each experiment was replicated three times.

### 2.4. Western Blot

Total proteins from mice kidneys and HK-2 cells were extracted, and equal amounts of protein were separated onto 10% SDS-PAGE and transferred to PVDF membranes (Millipore, MA). Then, the membranes were incubated with primary antibodies overnight at 4°C, followed by 1 h at 37°C with the appropriate HRP-conjugated secondary antibodies. Primary antibodies including REDD1 (1 : 500, sc-376671, Santa Cruz, CA, USA), TXNIP (1 : 1,000, #14715, CST, USA), Cleaved Caspase-3 (1 : 1000, #9661, CST, USA), Nox4 (1 : 500, 14347-1-AP, Proteintech, China), Bax (1 : 1000, 50599-2-Ig, Proteintech, China), Bcl-2 (1 : 500, 26593-1-AP, Proteintech, China), E-cadherin (1 : 1,000, 20874-1-AP, Proteintech, China), and *α*-SMA (1 : 1000, 14395-1-AP, Proteintech, China). Subsequently, the Odyssey Fc System (LI-COR, USA) was utilized to scan. In order to perform coimmunoprecipitation, samples were lysed on ice with lysis buffer for 30 mins and then centrifuged at 12,000 rpm for 20 mins. The supernatant was treated with the primary antibodies at 4°C overnight, followed by the addition of protein A/G sepharose (Santa Cruz, Dallas, TX) for 3 h. After three washes with homogenization buffer, the beads were boiled with a 6·buffer for 5 mins followed by SDS-PAGE and western blot with appropriate antibodies.

### 2.5. Apoptosis Assay

The RTEC apoptosis was measured with a terminal deoxynucleotidyl transferase-mediated dUTP Nick end labeling (TUNEL) test kit, as directed by the manufacturer. The number of apoptotic cells in each sample was counted in six distinct fields (×400) and averaged. The number of apoptotic cells was detected utilizing an annexin V/PI apoptosis detection kit as directed by the manufacturer (BD Biosciences, Franklin Lakes, NJ). HK-2 cells were resuspended in 1 × binding buffer and treated for 10 mins under dim light and at room, ambient temperature with 5 ml annexin V (conjugated with fluorescein isothiocyanate), 5 ml PI, and 5 ml FITC annexin V. A flow cytometer (BD Immunocytometry Systems, Franklin Lakes, NJ) was then used to analyze cell fluorescence.

### 2.6. Mitochondrial ROS Detection

MitoSOX (Sigma) was used to detect the generation of mitochondrial ROS. After culturing for 48 h with specific experimental stimuli, HK-2 cells were incubated with MitoSOX (5 *μ*M) at 37°C for 30 mins. Afterward, PBS was used to wash the HK-2 cells before being photographed with confocal microscopy (Leica, Germany).

### 2.7. Flow Cytometric Analysis of Intracellular ROS

CM-DCHF-DA (Invitrogen) evaluated the intracellular formation of ROS in HK-2 cells. The cells were cultured in PBS supplemented with 10 *μ*M DCHF-DA for half an hour at 37°C. Subsequently, cells were assessed by flow cytometry (BD Biosciences, Franklin Lakes, NJ).

### 2.8. Immunofluorescence Assay

DN patients' and mice's frozen renal tissue sections and HK-2 cells were fixed with precooling 4% paraformaldehyde for 30 mins, followed by 10% goat serum block for half an hour at 37°C. Specific primary antibodies incubated the cells in PBS overnight at 4°C, followed by treatment with FITC-conjugated secondary antibodies for 2 h at 37°C. Subsequently, nuclei were stained with DAPI. Finally, the sections and cells were assessed by fluorescence microscope (Olympus, Japan).

### 2.9. Tissue Histology and Immunohistochemistry

Three *μ*m thick paraffin-embedded kidney sections were deparaffinized in xylene, and graded ethanol was used for dehydration. Then, after rehydration, antigen repair, and blocking with goat serum, the renal tissue sections were used as primary antibodies against REDD1, TXNIP, and AQ-1 overnightat 4°C. Afterward, the sections were treated with secondary antibodies tagged with biotin for 30 mins at 37°C. Next, a DAB kit was used for immunostaining and finally observed with a light microscope.

### 2.10. Statistical Analyses

Data are presented as means ± SD. Statistical differences were assessed using two-tailed multivariate ANOVA for repeated measures. *P* < 0.05 was statistically significant.

## 3. Results

### 3.1. Clinical Characteristics

Forty patients with different stages of DN and 20 controls (healthy controls, 10 patients; type 2 diabetes controls, 10 patients) were included in the present study. Patients' clinical and biological characteristics are summarized in Table 1. Patients with DN stage IV had a longer history of diabetes and a higher HbA1c than patients with DN stages I, II, or III (*P* < 0.05). The hemoglobin level was significantly lower in patients with DN stage IV than in controls and other stages of DN (*P* < 0.05). Serum creatinine gradually increased with DN progression and while eGFR decreased with DN progression. Blood UA, *β*2MG, cystatin C, TC, TG, and LDL were considerably higher in patients with DN stage IV than in controls and other classes of DN (all *P* < 0.05). Histological features of patients with different DN stages and controls are shown in Table 1. 60% of patients with DN stage IV had an IFTA score 3 and an interstitial inflammation score 2.

### 3.2. REDD1 Is Increased in the Kidneys of Patients with DN and the Correlation Analysis of REDD1 with Clinical Indicators

**Figure 1:**
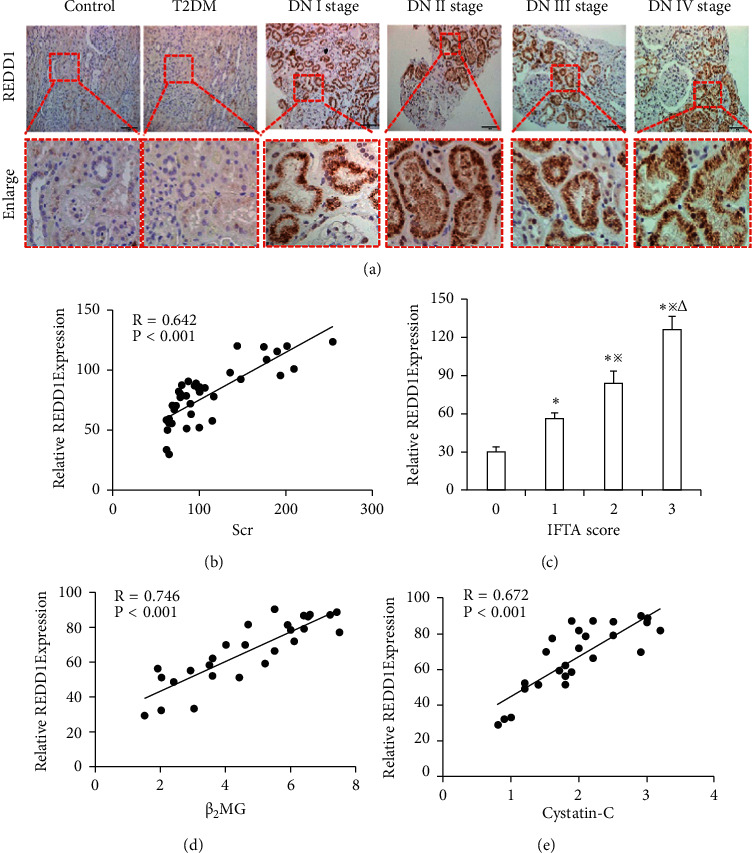
Expression of REDD1 in the kidneys of patients with DN and the correlation analysis of REDD1 with clinical indicators. (a) Immunohistochemical staining analysis of the expression of REDD1 in diabetic nephropathy biopsies (scale bar, 100 *μ*m, *n* = 6). (b) Correlation between Scr and the expression level of REDD1 in DN patients (*n* = 40). (c) The expression level of REDD1 according to IFTA score (*n* = 40). (d, e) Correlation between *β*2MG or cystatin C and the expression level of REDD1 in DN patients (*n* = 27). Values are expressed as means ± SD. ^*∗*^*P* < 0.05*versus* IFTA score 0 group; ^※^*P* < 0.05*versus* IFTA score 1 group; ^△^*P* < 0.05*versus* IFTA score 1 group.

In order to validate the expression level of REDD1 in the kidney, immunohistochemical analysis was performed on patients with pathologically diagnosed DN. In control tissues (surgical removal of normal and type 2 diabetes kidney tissue), a low level of REDD1 was seen in the kidneys. In contrast, significantly stronger expression of REDD1 was detected in the kidneys of patients with different DN stages, especially in the renal tubules (Figure 1(a)). We next evaluated the correlation between REDD1 and clinical indicators in the different DN subgroups. As shown in (Figure 1(b)), there was a substantial positive connection between Scr and the degree of REDD1 expression in DN patients. We also found that the expression of REDD1 positively correlated with *β*2MG and cystatin C in DN patients with normal serum creatinine (Figures 1(d) and 1(e)). Moreover, the expression level of REDD1 gradually increased, like the IFTA score (Figure 1(c)).

### 3.3. HG Increases the Expression of REDD1 in the Kidneys of Diabetic Mice and HK-2 Cells

**Figure 2:**
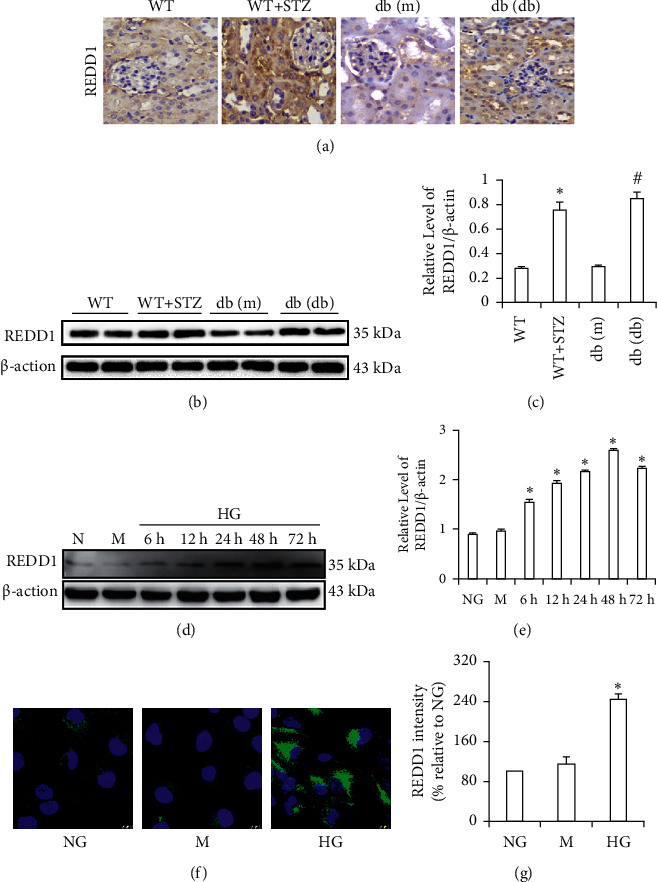
Expression of REDD1 in the kidneys of diabetic mice and HK-2 cells. (a) Immunohistochemical staining analysis of the expression of REDD1 in db/db mice and STZ-induced diabetic mice (scale bar, 50 *μ*m). (b, c) Western blot of REDD1 in the renal tissues. (d, e) Western blot of REDD1 in HK-2 cells were treated with high glucose (30 mM) at varying time points. (f, g) Immunofluorescence analysis of REDD1 expression in HK-2 cells. Values are expressed as means ± SD (*n* = 5 per group). NG: normal glucose group, 5.6 mM D-glucose; M: hypertonic control group, NG + 24.4 mM mannitol; HG: high glucose group, 30 mM D-glucose. ^*∗*^*P* < 0.05*versus* NG.

We next detected the expression of REDD1 in diabetic mice kidneys. The immunohistochemistry examination revealed that the REDD1 expression was considerably higher in the kidneys of streptozotocin- (STZ-) induced diabetic mice and db/db mice than in the control mice (Figure 2(a)). As shown in Figures 2(b) and 2(c), the protein levels of REDD1 were significantly increased in db/db mice and STZ-induced diabetic mice. Meanwhile, HK-2 cells were incubated with HG *in vitro* to determine the function of HG in the production of REDD1 and western blot determined REDD1 protein levels at different time points. As shown in Figures 2(d) and 2(e), the expression of REDD1 protein levels in HG-stimulated HK-2 cells was time-dependent, starting to increase after 6 h and peaking at 48 h. HG stimulated high expression of REDD1 was further proved *via* immunofluorescence (Figures 2(f) and 2(g)). In addition, there was no statistical difference between the NG and mannitol groups in the protein content of REDD1 of HK-2 cells.

### 3.4. REDD1 Deficiency Attenuates Apoptosis in Diabetic Mice

**Figure 3:**
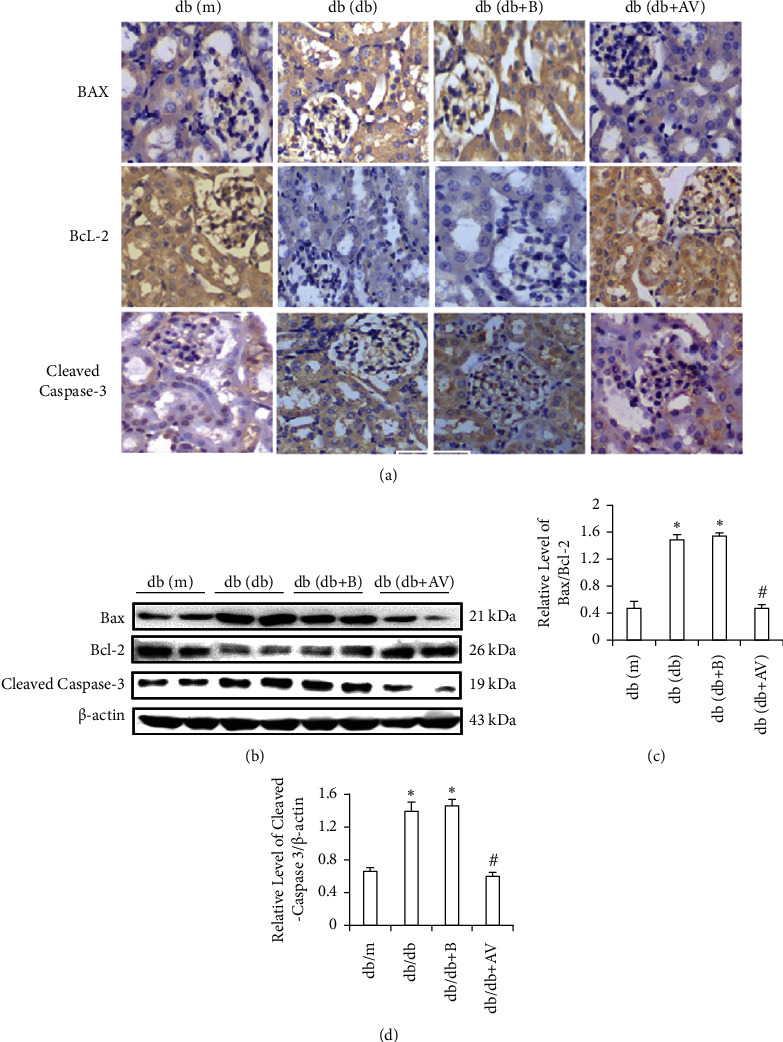
REDD1 deficiency attenuates apoptosis in diabetic mice. (a) The expression of Bax, Bcl-2, and Cleaved Caspase-3 was examined by immunohistochemistry (scale bar, 50 *μ*m). (b, c, d) The expression of Bax, Bcl-2, and Cleaved Caspase-3 was determined by western blot analysis. Values are expressed as means ± SD (*n* = 5 per group). ^*∗*^*P* < 0.05*versus* db/m group; ^#^^*∗*^*P* < 0.05*versus* db/db and db/db + *B* groups.

Exploring the role of REDD1 in kidney cell apoptosis in diabetic mice, we examined the indicators of apoptosis by immunohistochemical staining. As shown in Figure 3(a), Bax and Cleaved Caspase-3 were substantially elevated in db/db mice compared with db/m mice, whereas Bcl-2 was considerably downregulated, although REDD1 loss completely reversed these alterations. As shown in Figures 3(b)–3(d), the findings of the western blot revealed that the Bax/Bcl-2 ratio and Cleavage Caspase-3 were extraordinarily upregulated in diabetic mice, whereas REDD1 deficiency markedly reversed these changes. These results indicate that REDD1 deficiency could attenuate apoptosis in kidney cells of diabetic mice.

### 3.5. REDD1 Deficiency Suppresses EMT of RTEC in Diabetic Mice

**Figure 4:**
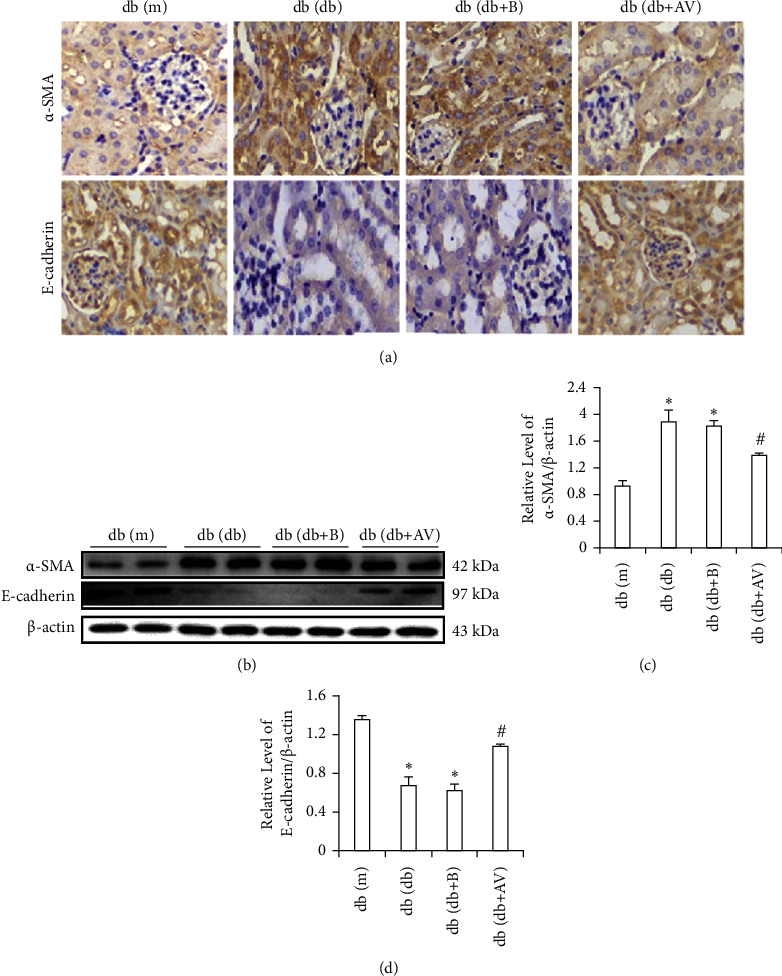
REDD1 deficiency suppresses EMT of RTEC in diabetic mice. (a) Immunohistochemistry examined the expression of *α*-SMA and E-cadherin (scale bar, 50 *μ*m). (b, c, d) The expression of *α*-SMA and E-cadherin was determined by western blot analysis. Values are expressed as means ± SD (*n* = 5 per group). ^*∗*^*P* < 0.05*versus* db/m group; ^#^*P* < 0.05*versus* db/db and db/db + *B* groups.

The role of REDD1 deficiency in the suppression of EMT in db/db mice was determined by appraising the expression of *α*-SMA and E-cadherin. Immunohistochemical and western blot assays revealed significantly higher expression of the mesenchymal marker *α*-SMA in db/db mice than in db/m mice (Figures 4(a)–4(c)). The expression of E-cadherin was opposite to *α*-SMA detected by immunohistochemistry and western blot (Figures 4(a)–4(c)). However, REDD1 deficiency reversed these changes (Figures 4(a)–4(c)). These results suggest that REDD1 deficiency could suppress EMT of RTEC in diabetic mice.

### 3.6. Knockdown of REDD1 Attenuates HG-Induced Apoptosis in HK-2 Cells

**Figure 5:**
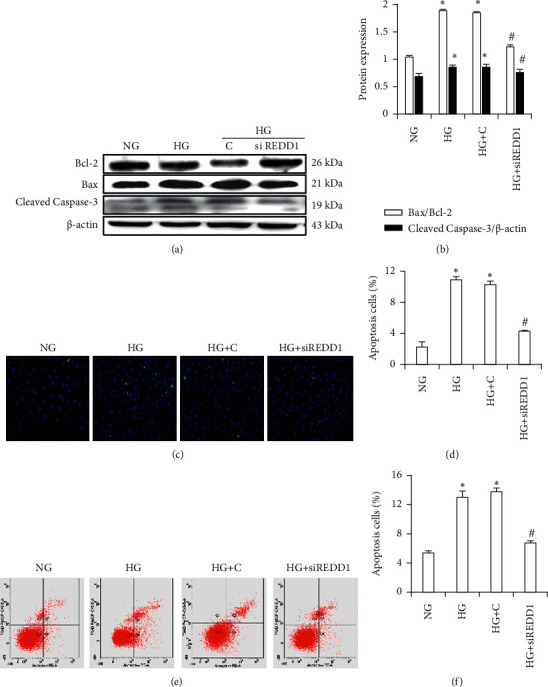
Knockdown of REDD1 inhibits HG-induced apoptosis in HK-2 cells. (a, b) Western blot of Bax, Bcl-2, and Cleaved Caspase-3 in HK-2 cells. (c, d) Apoptosis of HK-2 cells were analyzed by TUNEL assay. (e, f) Apoptosis rates of HK-2 cells were detected by flow cytometry analysis. Values are expressed as means ± SD (*n* = 6 per group). NG: 5.6 mM D-glucose; HG: 30 mM D-glucose; HG + C: 30 mM D-glucose plus scramble. HG + siREDD1: 30 mM D-glucose plus REDD1 siRNA. ^*∗*^*P* < 0.01*versus* NG; ^#^*P* < 0.05*versus* HG.

In order to verify the role of REDD1 in HG-induced apoptosis in HK-2 cells, after HG stimulation, cells were transfected with a REDD1 siRNA plasmid or a control vector, and then cell apoptosis was accurately measured. Following 48 h of HG treatment, cell apoptosis was assessed. Western blot results showed that the inhibition of REDD1 greatly attenuated Bax and Cleaved Caspase-3 in the HG group but dramatically boosted the protein level of Bcl-2 (Figures 5(a)-5(b)). As indicated in (Figures 5(c)–5(f)), REDD1 siRNA plasmid effectively reduced the HG-mediated elevation in apoptotic cell percentage in TUNEL assay and flow cytometry analysis results. Collectively, these data endorse the idea that REDD1 is involved in regulating renal tubular cell apoptosis triggered by HG.

### 3.7. Knockdown of REDD1 Mediates HG-Induced EMT in HK-2 Cells

**Figure 6:**
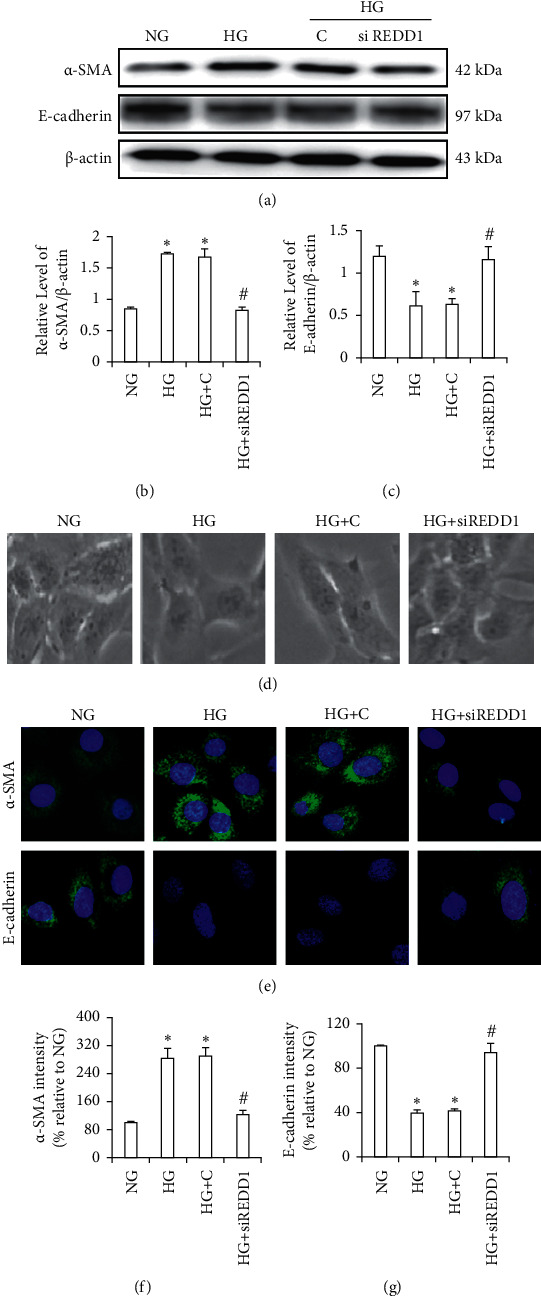
Knockdown of REDD1 inhibits HG-induced EMT in HK-2 cells. (a, b, c) Western blot of *α*-SMA and E-cadherin in HK-2 cells. (d) The inverted microscope was used to observe the morphological changes of HK-2 cells cultivated under various circumstances. (e, f, g) Immunofluorescence was used to detect the expression of *α*-SMA and E-cadherin in HK-2 cells. Values are expressed as means ± SD (*n* = 6 per group). NG: 5.6 mM D-glucose; HG: 30 mM D-glucose; HG + C: 30 mM D-glucose plus scramble. HG + siREDD1: 30 mM D-glucose plus REDD1 siRNA. ^*∗*^*P* < 0.01*versus* NG; ^#^*P* < 0.05*versus* HG.

To validate the role of REDD1 in the HG-induced EMT in renal tubular cells, we first observed the cell morphology. HK-2 cells growing in NG seemed to have a classic epithelial cuboidal shape with a cobblestone morphology; however, after incubation with HG, the cells became longer, lower adherent, and lacked their apical-to-basal polarity. Nevertheless, REDD1 knockdown corrected the morphological alterations induced by HG (Figure 6(d)). Western blot showed that HG-incubated HK-2 cells with REDD1 siRNA plasmid revealed a remarkable augment in E-cadherin and a decline in *α*-SMA expression compared with the NG group (Figures 6(a)–6(c)). Furthermore, we also validated these findings by immunofluorescence (Figures 6(e)–6(g)). Together, these data endorse the idea that REDD1 gets involved in regulating renal tubular cell EMT triggered by HG.

### 3.8. Knockdown of REDD1 Ameliorates HG-Induced Oxidative Stress in HK-2 Cells

**Figure 7:**
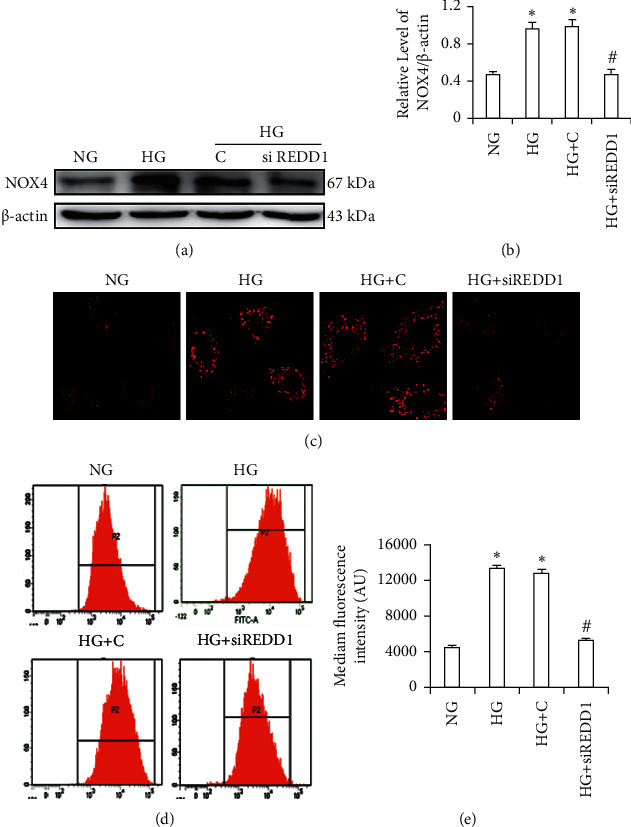
Knockdown of REDD1 inhibits HG-induced ROS production in HK-2 cells. (a, b) Western blot detected NOX4. (c) Mitochondrial ROS was detected by the confocal microscope. (d, e) Intracellular ROS was detected by flow cytometry. Values are expressed as means ± SD (*n* = 6 per group). NG: 5.6 mM D-glucose; HG: 30 mM D-glucose; HG + (C) 30 mM D-glucose plus scramble. HG + siREDD1: 30 mM D-glucose plus REDD1 siRNA. ^*∗*^*P* < 0.05*versus* NG; ^#^^*∗*^*P* < 0.05*versus* HG.

In order to explore the role of REDD1 suppression in HG-induced ROS, our experiment transfected HK-2 cells with REDD1 siRNA plasmid and then treated them with HG for 2 days. The ROS was monitored by fluorescent probes. HG could obviously induce the generation of total ROS and mitochondrial ROS in HK-2 cells. Nevertheless, the transfection of REDD1 siRNA plasmid into HK-2 cells dramatically attenuated HG-induced ROS levels (Figures 7(c)–7(e)). At the same time, under HG conditions, transfection with REDD1 siRNA plasmid considerably reduced the protein level of Nox4 (Figures 7(a)-7(b)). These data endorse the idea that hindering REDD1 may partly ameliorate oxidative stress induced by HG.

### 3.9. Knockdown of REDD1 Attenuates Renal Tubular Cells Dysfunction Partly through TXNIP

**Figure 8:**
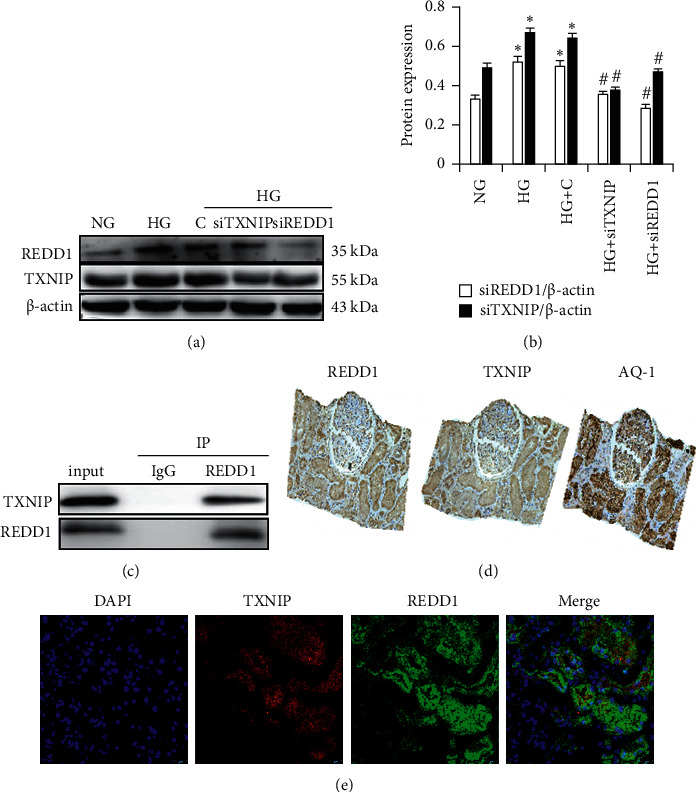
The relationship between REDD1 and TXNIP. (a, b) The expression of REDD1, TXNIP, and *β*-actin was analyzed by Western blot in HK-2 cells. (c) HG-induced endogenous REDD1/TXNIP complex was detected by Co-IP. (d) Immunohistochemical staining analysis of the expression of REDD1, TXNIP, and AQ-1 in diabetic nephropathy biopsies. (e) Double immunofluorescence labeling method analysis of the expression of REDD1 and TXNIP in the kidneys of diabetic nephropathy biopsies. Values are expressed as means ± SD (*n* = 6 per group). NG: 5.6 mM D-glucose; HG: 30 mM D-glucose; HG + (C) 30 mM D-glucose plus scramble. HG + siREDD1: 30 mM D-glucose + REDD1 siRNA. HG + siTXNIP: 30 mM D-glucose + TXNIP siRNA. ^*∗*^*P* < 0.01*versus* NG; ^#^*P* < 0.05*versus* HG.

While these results showed that REDD1-regulated ROS was important signaling intermediates for apoptosis and EMT, we do not fully understand how REDD1 regulates cellular ROS production. Continuous pathological sections of the kidney of DN biopsy were used for immunohistochemical staining of REDD1, TXNIP, and renal proximal tubule marker protein (AQP-1). The findings revealed that the expression sites of REDD1 and TXNIP were basically identical, whereas they were significantly enhanced in the proximal tubular cells (Figure 8(d)). We further confirmed that REDD1 and TXNIP were coexpressed in DN by immunofluorescence double-labeling (Figure 8(e)). In summary, there may be interactions between REDD1 and TXNIP. As illustrated in Figures 8(a)-8(b), the TXNIP expression was enhanced in the HG group, which was corrected by the REDD1 knockdown. Additionally, we focused on the impact of TXNIP on the REDD1 expression in the HG group, and the results proved that TXNIP depletion massively reduced HG-induced REDD1 synthesis. Furthermore, using co-IP, the connection between endogenous REDD1 and TXNIP was clearly verified. Most notably, the endogenous REDD1/TXNIP complex was activated under HG circumstances (Figure 8(c)). In response to HG, endogenous REDD1 and TXNIP are generated and develop a robust biophysical complex.

### 3.10. Antioxidant Inhibits the Expression of REDD1

**Figure 9:**
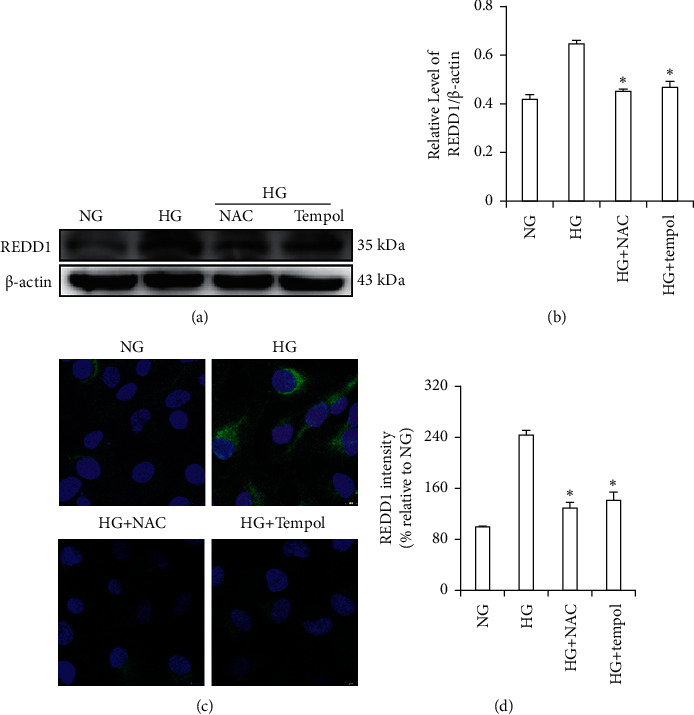
Effects of antioxidant interference on HG-induced REDD1 in HK-2 cells. (a, b) The expression of REDD1 and *β*-actin was analyzed by western blot in HK-2 cells. (c, d) Immunofluorescence analysis of the expression of REDD1 in HK-2 cells. Values are expressed as means ± SD (*n* = 6 per group). NG: 5.6 mM D-glucose; HG: 30 mM D-glucose; HG + NAC: 30 mM D-glucose + N-acetylcysteine (5 mM); HG + Tempol: 30 mM D-glucose + Tempol (100 nM). ^*∗*^*P* < 0.05*versus* HG.

We wanted to know the effect of antioxidants on the REDD1 expression. The samples were incubated with NAC and Tempol for 48 h. Our results indicated that REDD1 protein levels increased in HG conditions more than in NG conditions. However, NAC treatment decreased the amount of REDD1 protein levels compared with the HG group, and the effect of Tempol on REDD1 was similar to the NAC group (Figures 9(a)-9(b)). In addition, we used immunofluorescence to determine the effect of the HG concentration and antioxidant agents' treatment on the REDD1 expression in HK-2 cells. The results were comparable to the western blot (Figures 9(c)-9(d)).

## 4. Discussion

RTEC apoptosis and EMT participate in the pathogenesis of DN [27, 28]. The underlying biological process, however, has still not been fully elucidated. REDD1 is a potential therapeutic target in many ways of chronic diseases. The primary goal of this investigation is to determine if REDD1 inhibition may serve as a useful therapeutic intervention for DN and whether RTEC is implicated in the pathological process of DN. The current work aims to determine the influence of REDD1 on apoptosis and EMT in DN and understand probable biologic mechanisms.

In the current research, we discovered the REDD1 expression in the kidneys of DN patients utilizing immunohistochemical labeling. Compared with the control group, REDD1 staining was extremely increased in the RTEC of kidneys of DN patients. Meanwhile, our results showed that the REDD1 expression was dramatically boosted with the aggravation of renal pathology in DN patients. Moreover, we observed that the expression of the REDD1 levels was considerably higher in individuals with advanced DN and those with a higher IFTA score. Although serum creatinine is a traditional biomarker, it is not very accurate because its values do not boost up until the GFR is lowered to less than 50% of normal [29]. Cystatin C and *β*2MG, as potential markers of renal injury, have higher accuracy in diagnosing DN than serum creatinine [30, 31]. Meanwhile, they are also a classic biomarker of renal tubulointerstitial injury [32, 33]. In our study, we detected REDD1 positively correlated with *β*2MG and cystatin C in DN patients with normal serum creatinine. The above results indicated that REDD1 may play a pivotal part in the RTEC injury of DN. Subsequently, *in vivo* and *in vitro*, the results of this experimental study showed that REDD1 expression was upregulated in diabetic/hyperglycemic situations. Compared with the control group, silencing REDD1 alleviated HG-induced ROS production, as shown by the ameliorated renal epithelial tubule cell apoptosis and EMT. As a result of these observations, it is concluded that REDD1 is implicated in the pathogenesis of DN.

The cell death shown in RTEC injury, which is an excellent feature of DN, is characterized by apoptosis. Accumulating evidence demonstrates that the inhibited REDD1 expression not only induced apoptosis of human umbilical vein endothelial cells after exposure to LPS, but also noticeably alleviated PM2.5-induced BEAS-2B cell apoptosis [21, 34]. A study reported that REDD1 overexpression is sufficient to promote neuronal apoptosis [35]. On the contrary, REDD1 overexpression protects against the occurrence and progression of heart failure after myocardial infarction by attenuating apoptosis [36]. Facing different cell types and responding to diverse stressors, REDD1 shows a bifunctional biological function as an antiapoptotic or proapoptotic factor. During our experiments, cellular apoptosis was considerably increased in the kidneys of diabetic mice. Interestingly, REDD1 deficiency alleviated this. *In vitro*, the results showed that HG boosted HK-2 cell apoptosis, along with the adjustment of apoptosis-related protein. In the meantime, we also found that transfection with REDD1 siRNA plasmid enormously relieved HK-2 cell apoptosis induced by HG and adjusted apoptosis-related proteins. Based on these data, we postulate that REDD1 suppression has antiapoptotic effects in preventing HK-2 cell apoptosis induced by HG. As a result, interfering REDD1 is essential in protecting RTEC against HG-induced apoptosis.

Tubulointerstitial fibrosis is the major pathogenic change driving the progression of DN, whereas RTEC transdifferentiation into myofibroblasts (i.e., EMT) is remarkable in tubulointerstitial fibrosis. Numerous experiments have confirmed that HG is linked to a variety of structural and functional changes in RTEC and that podocyte EMT is also one of the characteristic lesions of DN [37, 38]. Therefore, EMT is the key process underlying HG-induced DN [28]. Moreover, our forepassed research has obviously confirmed that the inhibition of thioredoxin-interacting protein (TXNIP) and NLRP3 inflammasome ameliorate HG-induced EMT in RTEC [26, 39]. In our research, we investigated the role of REDD1 siRNA plasmid in EMT treated by HG *in vitro* or *in vivo*. Our study revealed that, in HK-2 cells stimulated with a high glucose level, the *α*-SMA expression increased, whereas the E-cadherin expression dropped. The knockdown of REDD1 inhibited HG-induced EMT. These findings show that REDD1 may play a role in renal tubular cell EMT induced by HG.

The activation of oxidative stress is pivotal in the apoptosis and EMT of DN [40,41]. HG-induced oxidative stress may be a crucial originator of RTEC apoptosis and EMT. The accumulation of ROS participates in RTEC injury. ROS production is implicated in RTEC apoptosis and EMT induced by HG [26, 42]. Thieme et al. found that REDD1 dramatically upregulated the ROS level in TP63-null fibroblasts [43]. Recent experiments have straightforwardly shown that REDD1 is involved in the modulation of redox status. For example, through modulating autophagic flow, REDD1 has been proven to sustain cellular ROS generation and adaptability to energy demand [44]. Furthermore, Qiao et al. have claimed that REDD1 absence causes neoplastic transformation by upping ROS-cleansing NADPH levels, which skews cellular metabolism [45]. On the contrary, through mediating ROS-induced destruction of the antioxidant transcription factor NRF2, REDD1 can contribute to the pathophysiology of DN [46]. Presently, there is no compelling proof that REDD1 plays a massive part in HG-induced oxidative stress. The generation of ROS in HK-2 cells was examined, and the results showed that knockdown of REDD1 diminished the formation of ROS following HG stimulation. Moreover, the level of NOX4 was upregulated in RTEC cells incubated by HG. However, the knockdown of REDD1 reversed these changes. Uniformly, our data provide adamant evidence advocating the viewpoint that restriction of REDD1 guards against HG-induced RTEC apoptosis and EMT maybe by the modulation of ROS.

In mammals, REDD1 operates as an upriver blocker of mTOR activity *via* the TSC1/2 complex [47, 48]. REDD1 plays a vital role in autophagy by inhibiting mTORC1 [49]. However, a study discovered that REDD1 is essential in activating autophagy through forming complexes with TXNIP to evoke ROS overexpression, apparently the mTORC1-independent mechanism [44]. Moreover, a recent study reported that REDD1 stabilizes *via* interactions with N-terminal truncated TXNIP [50]. TXNIP is a prooxidative molecule known to contribute to many different diseases, particularly DN [51]. TXNIP, as a natural inhibitor of thioredoxin, is a ubiquitously expressed protein significantly induced by HG [52]. Many studies have indicated that REDD1 potentially interacts with TXNIP to diminish ATG4B under oxidative responses. Increased REDD1/TXNIP complex generation was advantageous for ROS formation [44]. Nevertheless, a recent study showed that, through the mitochondrial mechanism, the REDD1/TXNIP complex promotes oxidative stress-induced apoptosis in nucleus pulposus cells [53]. The main conclusion of this work is that REDD1 and TXNIP form a combination to activate endogenous ROS generation in the presence of HG. Additionally, what is different from the previous report is that the REDD1/TXNIP complex activates apoptosis rather than autophagy [54]. A study has reported that NAC reduced the REDD1 protein level in the retina of diabetic mice [55]. Our previous reports have shown that antioxidant NAC suppressed HG-induced TXNIP expression [39]. Beyond that, NAC also could inhibit TXNIP production in nondiabetic kidney diseases [43]. In our study, we also authenticated that treatment with antioxidants, NAC and Tempol, effectively suppressed the expression of REDD1 induced by HG. Our data further demonstrate that REDD1 suppression against HG-induced HK-2 cell apoptosis and EMT were partly due to TXNIP rather than through mTORC1.

## 5. Conclusion

In summary, the suppression of REDD1 decreases HG-induced apoptosis and EMT in RTEC, according to our findings in this trial. This study also highlights that REDD1 and TXNIP form a biological complex aided by HG. The mitochondrial route is used by the REDD1/TXNIP complex to drive oxidative stress-induced RTEC apoptosis and EMT. Thus, considering the activity of the REDD1/TXNIP complex can be used as an extraordinary and probably useful treatment modality for DN prevention and therapy.

## Figures and Tables

**Table 1:** General characteristics of the control, diabetes, and DN groups. *n* = number. Data presented as means ± SD or *n* prevalence. Glu: blood glucose; HbA1c: glycosylated hemoglobin; Hb: hemoglobin; Alb: serum albumin; Scr: serum creatinine; eGFR: estimated glomerular filtration rate; *β*2MG: *β*2-microglobulin; UA: uric acid; TC: total cholesterol; TG: total triglyceride; LDL: low-density lipoprotein; BMI: body mass index; ^*∗*^*P* < 0.05*versus* control group; ^※^*P* < 0.05*versus* diabetes group; ^#^*P* < 0.05*versus* DN stage I group; ^△^*P* < 0.05*versus* DN stage II group; ^&^*P* < 0.05*versus* DN stage III group (all one-way analysis of variance).

	Control group	Diabetes group	Diabetic nephropathy group			
			Stage I	Stage II	Stage III	Stage IV
Participants (*n*)	10	10	10	10	10	10
Age (years)	54 ± 7	55 ± 7	53 ± 8	54 ± 8	58 ± 10	60 ± 9
Sex (male)	5	5	4	6	5	6
Diabetes history (years)	—	11.8 ± 3.3	10.8 ± 3.8	12.3 ± 4.2	13.3 ± 5.1	14.8 ± 5.7^※#^
BMI (kg/m^2^)	24.2 ± 3.0	25.2 ± 2.7	24.3 ± 2.8	25.3 ± 2.7	26.9 ± 2.8	26.7 ± 2.7
Glu (mmo/l)	5.2 ± 0.6	8.6 ± 1.3^*∗*^	8.1 ± 1.1^*∗*^	8.3 ± 1.3^*∗*^	9.4 ± 1.5^*∗*^	10.1 ± 1.9^*∗*^
HbA1c (%)	4.8 ± 0.6	6.5 ± 0.8^*∗*^	6.6 ± 0.7^*∗*^	6.2 ± 1.1^*∗*^	6.6 ± 1.3^*∗*^	8.1 ± 1.6^*∗*^^※#△&^
Hb (g/L)	132 ± 12	133 ± 11	131 ± 14	127 ± 11	120 ± 10	113 ± 15*∗*^※#^
Alb (g/L)	39.8 ± 7.1	38.4 ± 7.0	37.3 ± 9.1	33.8 ± 5.6	30.3 ± 5.3	29.1 ± 5.6
Scr (mmo/l)	67.1 ± 13.3	66.6 ± 12.6	62.4 ± 10.2	105.3 ± 25.1^*∗*^^※#^	112.2 ± 30.6^*∗*^^※#^	276.8 ± 34.8^*∗*^^※#△&^
eGFR (ml/min/1.73 m^2^)	101.6 ± 9.7	100.2 ± 9.4	100.5 ± 9.4	80.3 ± 8.5^*∗*^^※#^	73.8 ± 7.8^*∗*^^※#^	45.6 ± 6.6^*∗*^^※#△&^
UA (mmo/l)	301.6 ± 76.8	300.6 ± 74.4	312.5 ± 74.3	382.5 ± 84.8^*∗*^^※#^	402.2 ± 89.2^*∗*^^※#^	500.5 ± 93.3^*∗*^^※#△&^
(mg/L)	0.8 ± 0.1	0.8 ± 0.2	1.1 ± 0.2	1.5 ± 0.3^*∗*^^※#^	1.8 ± 0.6^*∗*^^※#^	2.7 ± 0.8^*∗*^^※#△&^
(mmo/l)	2.2 ± 1.0	2.1 ± 1.0	2.4 ± 1.1	3.7 ± 1.5^*∗*^^※#^	4.3 ± 2.0^*∗*^^※#^	7.1 ± 4.1^*∗*^^※#△&^
TC (mmo/l)	3.7 ± 1.1	3.7 ± 1.2	4.0 ± 0.9^*∗*^^※^	4.3 ± 1.3^*∗*^^※#^	4.7 ± 1.1^*∗*^^※#△^	5.9 ± 2.0^*∗*^^※#△&^
TG (mmo/l)	0.9 ± 0.3	0.8 ± 0.3	1.4 ± 0.4^*∗*^^※^	1.9 ± 0.9^*∗*^^※#^	2.4 ± 1.1^*∗*^^※#△^	3.1 ± 1.9^*∗*^^※#△&^
LDL (mmo/l)	1.8 ± 0.5	1.9 ± 0.6	2.1 ± 0.5^*∗*^^※^	2.5 ± 1.1^*∗*^^※#^	3.6 ± 1.6^*∗*^^※#△^	4.1 ± 1.9^*∗*^^※#△&^
IFTA (%)						
0	9 (90%)	8 (80%)	5 (50%)	6 (60%)	2 (20%)	0
1	1 (10%)	2 (20%)	3 (30%)	2 (20%)	2 (20%)	2 (20%)
2	0	0	2 (20%)	1 (10%)	4 (40%)	2 (20%)
3	—		0	1 (10%)	2 (20%)	6 (60%)
Interstitial inflammation (%)						
0	10 (100%)	9 (90%)	7 (70%)	6 (60%)	5 (50%)	1 (10%)
1	0	1 (10%)	3 (30%)	3 (30%)	3 (30%)	3 (30%)
2	0	0	0	1 (10%)	2 (2.7%)	6 (60%)

## Data Availability

The data used to support the study's conclusions are included in the paper.
